# To what extent do potential conservation donors value community-aspects of conservation projects in low income countries?

**DOI:** 10.1371/journal.pone.0192935

**Published:** 2018-02-16

**Authors:** Amy R. Lewis, Richard P. Young, James M. Gibbons, Julia P. G. Jones

**Affiliations:** 1 School of Environment, Natural Resources and Geography, Bangor University, Gwynedd, Wales; 2 Durrell Wildlife Conservation Trust, Trinity, Jersey, Channel Islands; 3 Department of Life Sciences, Imperial College London, Silwood Park Campus, Ascot, Berkshire, United Kingdom; UNITED STATES

## Abstract

There is a major gap in funding required for conservation, especially in low income countries. Given the significant contribution of taxpayers in industrialized countries to funding conservation overseas, and donations from membership organisation, understanding the preferences of ordinary people in a high income country for different attributes of conservation projects is valuable for future marketing of conservation. We conducted a discrete choice experiment with visitors to a UK zoo, while simultaneously conducting a revealed preference study through a real donation campaign on the same sample. Respondents showed the highest willingness to pay for projects that have local community involvement in management (95% confidence interval £9.82 to £15.83), and for improvement in threatened species populations (£2.97 - £13.87). Both of these were significantly larger than the willingness to pay for projects involving provision of alternative livelihoods, or improving the condition of conservation sites. Results of the simultaneous donation campaign showed that respondents were very willing to donate the suggested £1 or above donation (88% made a donation, n = 1798); there was no effect of which of the two campaigns they were exposed to (threatened species management or community involvement in management). The small number of people who did not make a donation had a higher stated willingness to pay within the choice experiment, which may suggest hypothetical bias. Conservationists increasingly argue that conservation should include local communities in management (for both pragmatic and moral reasons). It is heartening that potential conservation donors seem to agree.

## Introduction

For the last few decades it has been widely recognised that conservation, while having national and global benefits, frequently brings local costs [1,2]. Given that areas of high biodiversity overlap with areas where poverty is widespread [3], it is increasingly argued that conservation should invest in human development alongside species and habitat based actions [4–6]. Delivering the dual goals of conservation and development has led to a mix of strategies to deliver conservation objectives; from strict protected areas (often with initiatives aimed at supporting local livelihoods) to community based conservation approaches which include local people in management [7,8].

Though money is not the only barrier to achieving conservation outcomes, there is a major gap between expenditure and need, which is most extreme in the tropics [9]. Every year the world spends around US$126 billion of official aid addressing global poverty and between US$8–16 billion addressing biodiversity loss [10,11], where there remains substantial unmet need [12]. Funding for biodiversity in developing countries include: domestic budget allocations (~US$11 billion); multilateral and bilateral aid (~US$4 billion); and philanthropy (including charitable trusts and conservation NGO funding, ~US$0.5–1 billion) [13]. The philanthropic element of biodiversity funding therefore represents approximately between 3% and 12% of current estimates [13] meaning public attitudes to what conservation projects should fund is important [14]. Understanding the preferences of donors for these different aspects of conservation projects such as involvement of local communities in management and decision making or providing alternative livelihoods, could help target and improve future marketing campaigns.

Various methods have been designed to measure the value people place on goods or services for which there is no current market [15]. Discrete choice experiments (referred to here as choice experiments) are a stated preference valuation technique where respondents are given a series of future scenarios and asked to make choices between them [16]. From these choices one can analyse an individuals’ preferences for the attributes that make up that scenario. Choice experiments are increasingly applied to questions important in conservation science. For example many studies have looked at the preference of potential donors for the management and protection of charismatic species [17–19]. However, these studies assume that individuals only value the outcome of a proposed intervention, not the structure by which it is implemented. Other studies have used stated preference techniques to value the preference local people place on the impact of different environmental management mechanisms on their communities and livelihoods [20–22]. A notable exception, however, is a recent paper that shows that potential foreign donors have preferences for distributive benefits of payments for ecosystem services to local people in Madagascar [23].

Despite the wide use of choice experiments, they may be prone to hypothetical bias, as respondents do not have to support their choices with real commitments. Few choice experiments are able to validate their findings through external validation with a real market due to the difficulty in identifying a market valuing the same attributes [24]. A recent systematic review by Rakotonarivo et al (2016) identifies 11 non market valuation choice experiment studies, published between 2003 and 2016, that attempt to validate their results [25]. Often such studies are laboratory based and use undergraduate students and use a binding choice (where they are obliged to part with a good/ real money) if a choice within the experiment is selected [26,27]. Only one study compared preferences made in a hypothetical choice with a revealed preference field study [28].

We use a choice experiment to explore the extent to which potential donors to a conservation project in Madagascar (visitors to Jersey Zoo, headquarters of the Durrell Wildlife Conservation Trust) value the various aspects of a conservation intervention (threatened species populations, community involvement in management, the condition of sites of conservation concern and investing in the provision of alternative livelihoods). We explore the characteristics of donors with a stated higher willingness to pay, and preferences for the various aspects of the conservation project. We also attempted to validate the results of the choice experiments by conducting a revealed preference trial where those entering the zoo were asked to make a small donation to a conservation project in Madagascar (the advertising alternating between a focus on threatened species populations or community involvement in management). This paper therefore adds to the very limited literature comparing a hypothetical choice experiment with field observation of revealed preferences. It also increases our understanding of the preferences of potential contributors to conservation projects among the general public; providing valuable marketing insights for conservation projects.

## Methods

### Case study

Bangor University Ethics Committee approved this research (CNS2015AL2). This study was carried out at Jersey Zoo, Chanel Islands, UK. Visitors to the zoo over the age of 18 were our target population. While it may be argued that zoo visitors have an above average interest in conservation, evidence suggests that zoos do reach a relatively representative cross section of society, and that the popularity of a zoo’s collection is more indicative of visitor numbers than socio-demographic indicators [29]. We therefore suggest that this sample provides useful information on the preferences of the general public in the UK, and probably industrialized countries more broadly, who could be easily targeted for donations from a conservation project.

Visitors to the zoo over the age of 18 were our target population. While it may be argued that zoo visitors have an above average interest in conservation, evidence suggests that zoos do reach a relatively representative cross section of society, and that the popularity of a zoo’s collection is more indicative of visitor numbers than socio-demographic indicators [29]. We therefore suggest that this sample provides useful information on the preferences of the general public in the UK, and probably industrialized countries more broadly, who may donate to conservation initiatives.

Jersey Zoo is run by the Durrell Wildlife Conservation Trust (hereafter abbreviated to Durrell). Durrell has been active in Madagascar for 30 years where they have high profile community-based conservation programmes and Jersey Zoo has populations of many of their target species from Madagascar and contains an exhibit modelled on a field site (the Menabe dry forest). Durrell runs regular fund-raising campaigns through the zoo to support their field programmes. At the time of this research, Durrell was planning a new campaign to generate more donor funding for conservation projects in Madagascar. This provided us with the opportunity to measure both stated preferences (using a choice experiment) and compare with revealed preferences (as measured through voluntary donations at the zoo entrance; the details of the campaign were altered weekly in an experimental set up).

Madagascar is a biodiversity hotspot [30] which has become one of the largest recipients of conservation funding among low income countries [31]. Since its independence, Madagascar has benefited from several hundred million US dollars of support for environment programmes [32]. A range of conservation approaches are in operation in Madagascar including threatened species protection [33], protecting habitats [34], providing alternative livelihoods [35,36], and involving the local community in the management of the project or intervention [37]. Of course many interventions will involve more than one approach. In 2003, the government of Madagascar committed to tripling the protected area network in Madagascar. This remains a primary conservation mechanism in Madagascar [38] but there has also been a significant increase in the number of community based conservation projects in Madagascar over the last 20 years [37], with over 1,000 community forest management sites alone [39,40].

### Choice experiment design

**Fig 1:**
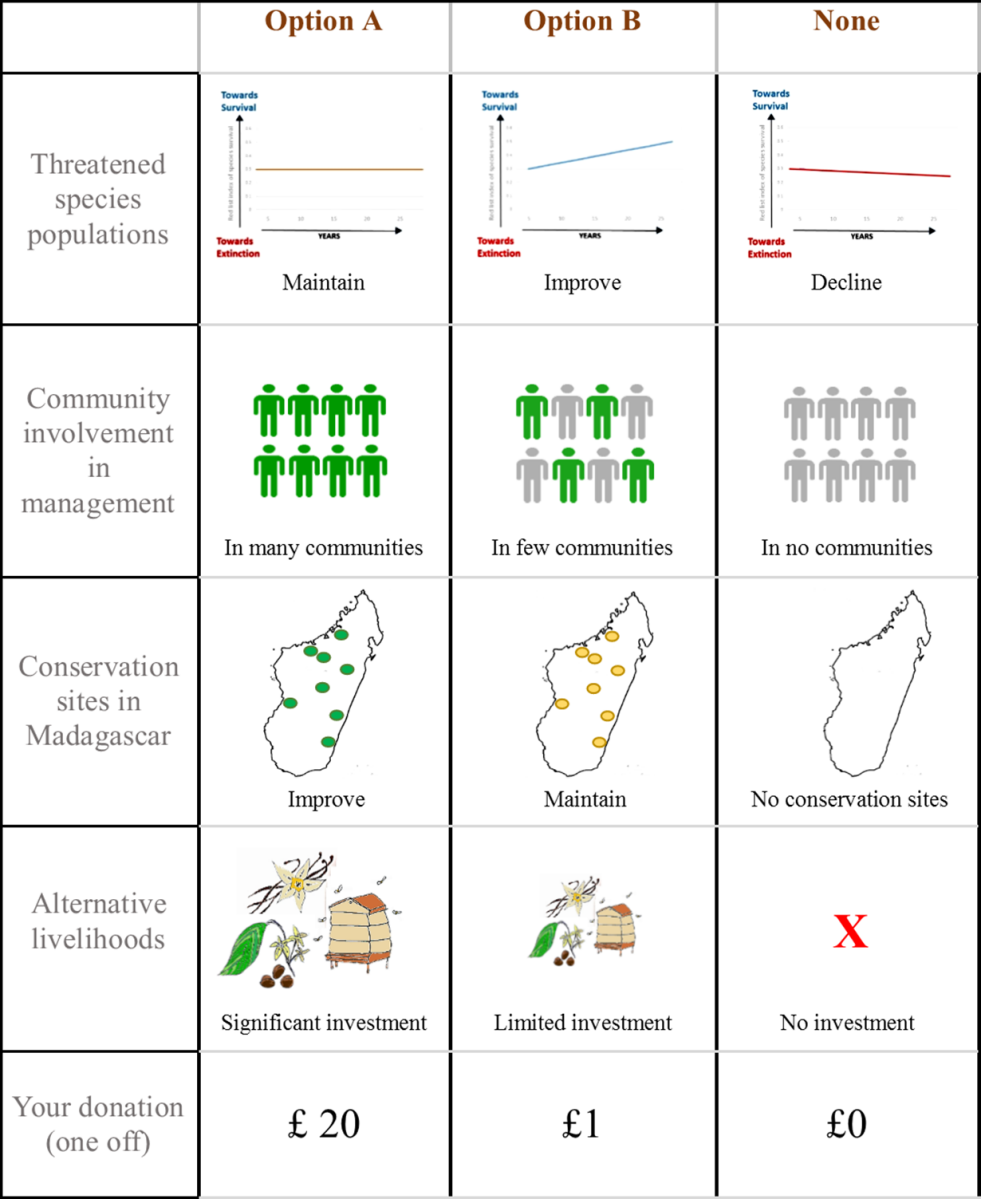
Sample choice task, where respondents were asked to select their preferred option.

The design of the choice experiment is based on hypothetical future conservation projects. The choice task was framed as a selection between different conservation management options that would require a financial contribution if selected. These future scenarios are described in terms of their attributes which are represented by levels (See Table 1 and Fig 1). In order to reduce the complexity we selected five attributes (four conservation attributes and a payment attribute to allow valuation in monetary terms). Each of the conservation attributes had three levels representing the potential levels of conservation interventions; a business as usual (BAU) scenario, where no further conservation measures are implemented; a moderate intervention of management and a substantial management intervention. The four conservation attributes were selected based on the literature and in consultation with conservation practitioners and aim to reflect the range of approaches to conservation. We wanted to have an equal number of community orientated attributed and ecological orientated attributes to enable us to associate these attributes with the revealed preference campaigns. The payment vehicle was determined as a one-off donation to enable us to validate our results with the real donations, and it was decided to include £1 as one of the payment levels, to match the real suggested donation, though other studies suggest the payment vehicle could be increased taxation or an addition to a utility bill [41].

**Table 1:** Conservation management approaches Madagascar, their attributes and levels used in the choice experiment and the validation method used in revealed preference study.

*Attribute*	*Definition*	*Management levels*	*Validation method*
*Threatened species populations*	The extent to which the conservation project’s focus is improving or maintaining populations of threatened species.	BAU: Population declines	Visitors were asked to make a £1 donation for a conservation project in Madagascar (focused on threatened species).
		Low: Maintain current populations	
		High: Population increases	
*Community involvement in management*	The extent to which local people are trained and empowered to protect their local environment.	BAU: In no communities	Visitors were asked to make a £1 donation for a conservation project in Madagascar (involving local communities in management).
		Low: In few communities	
		High: In many communities	
*Site focus*	The extent to which the conservation project improves or maintains the condition of conservation sites.	BAU: No conservation activity	None
		Low: Maintain the field sites	
		High: Improve the field sites	
*Provision of alternative livelihoods*	The extent to which the conservation project invests in supporting alternative livelihoods for local communities.	BAU: No investment	None
		Low: Limited investment	
		High: Significant investment.	
*Donation (one off)*	A one-off payment to support the project campaign.	£0, £1, £5, £20, £50	Real donation of £1 or more to either Marketing campaign

Note: Each attribute has three levels of conservation interventions including a business as usual scenario (BAU). Payment levels were determined in the pilot study.

The first of the choice experiment attributes was a focus on threatened species populations. This was explained with images of Malagasy threatened species: the Alaotran gentle lemur; the Madagascar pochard; the Flat-tailed tortoise; and the Madagascar giant jumping rat. The attribute included the increase, maintenance or decline of these threatened species. The second attribute concerned the extent to which local communities are explicitly involved in the management the conservation project. This includes training and empowerment of local individuals and reflects the way in which Durrell operate in many of their sites [42,43]. The third attribute focused on improving the condition of protected areas across Madagascar, levels included no sites, maintenance of sites or an improvement in the condition. The fourth attribute was the investment in alternative livelihoods for local communities as part of the conservation project. Examples given were: growing coffee, growing vanilla and providing bee keeping equipment. In addition, a payment attribute was selected. This was described as a one-off donation to contribute to the conservation project and ranged from £0 to £50. Note that the zero payment option was only included in the BAU option due to the fact that the management options all required payment.

The attributes are clearly not completely stand-alone; for example, threatened species populations and the condition of conservation sites are closely linked as the condition of sites will influence threatened species populations. However a project may focus on species-based actions (e.g. enforcing anti-hunting laws, removing invasive predators) without a focus on habitat so we treat these as separate attributes. Similarly, the provision of alternative livelihoods does not inherently involve active community participation and decision making in a conservation project [44] which is why these are included as separate attributes. In order to make sure respondents understood the task, each attribute, and the vocabulary used in the survey, a pilot study was conducted (n = 14). This enabled us to further refine the design and to define the choke point for the payment attribute, where individuals would not be willing to pay above a certain amount [45]. In order to make sure respondents understood both the task, each attribute and the vocabulary used in the survey a pilot study was conducted (n = 14). This enabled us to further refine the design and to define the choke point for the payment attribute, where individuals would not be willing to pay above a certain amount [45].

A large number of unique conservation management scenarios can be constructed from this number of attributes and levels. Sawtooth software (V.3.2) and fractional factorial design techniques were used to obtain a choice experiment design, which consisted of only the main effects. This resulted in 36 pair-wise comparisons of alternative management scenarios which were randomly blocked to 4 choice sets, each with 9 choice tasks. Each choice task contained two management scenarios and the BAU scenario with the corresponding zero donation. The BAU option is necessary to achieving welfare measures that are consistent with demand theory [46]. If the BAU is omitted respondents may be forced to choose an option that they do not have any reference for and therefore could overestimate willingness to pay. Further details on the design of choice experiments can be found in Hanley et al (1988) [47].

### Choice experiment data collection

The survey was conducted during July and August 2016 with face-to-face interviews and recorded on Android phones using Open Data Kit (ODK) [48]. These were conducted over a 4 week period including a week before the school holidays began. Interviews were carried out by ARL and one research assistant. We aimed to obtain a representative sample of adult paying footfall through the ticket gates, visitors were approached opportunistically after entering the zoo, and only 11% of those approached refused to participate in the study. We do not believe respondents associated the choice experiment interview with the request for a donation at the gate as these were separate processes; one a formal zoo fund-raising activity and the other research conducted by researchers from a university. Interviews lasted 20 minutes on average and no longer than 30 minutes.

The choice experiment was introduced by explaining each of the attributes, as well as the financial constraints in delivering these conservation scenarios, and individuals were presented with a practice choice task and time to ask questions. Throughout completion of the choice tasks respondents were reminded to consider their household budgetary constraints. Following the choice tasks we asked a series of short questions to collect socio-economic characteristics such as age, income and previous donations to charities (and whether these charities focused on humanitarian work or were wildlife focused). These were included as explanatory variables to explore heterogeneity in preferences, as well as to analyse the sample against paying visitors to the zoo.

### Revealed preference design and data collection

In addition to the choice experiment, we wanted to compare the preferences for the attributes based on a real conservation campaign. This was designed in collaboration with the Durrell marketing department, during its 2016 campaign to raise money for their conservation projects in Madagascar. The campaign was on the same population as the choice experiment sample and ran during the same period as the choice experiment. Visitors were asked at the tills for an additional one-off donation to raise money for a conservation project in Madagascar (all money did indeed go directly to support Durrell’s work in Madagascar). The experimental campaign ran for four weeks, split equally between threatened species management and community involvement in management in Madagascar (alternating weekly, see Table A in S2 Appendix).

The two campaigns were presented to visitors at the entrance to the zoo in the form of posters and leaflets. A £1 additional donation was asked for (though more could be given). The donation confirmation and amount was recorded within the till data which could then be extracted and linked to the choice experiment responses by scanning the till ticket bar code.

All visitors were therefore exposed to either campaign (approximately half to each of the two formulations: a focus on threatened species or community involvement in management). Our experiment was only able to run for a period of four weeks therefore the revealed preference results were limited to a subset of visitors to the zoo during that period (n = 1798).

A small sub-set of visitors (n = 244) then went on to complete the choice experiment. Unfortunately due to logistical constraints only some visitors were asked to give a donation, therefore not all those who completed the choice experiment had been asked to make a donation.

### Ethics and data management

The research was scrutinised and cleared under the Bangor University Research Ethics Framework. During the interviews we introduced ourselves and the task involved. Respondents were reminded that they could stop at any time without giving any explanation. The oral consent script (see S1 Appendix), ODK technical skills as well as interview techniques were practiced during the training period (1 week). We read a script explaining the purpose of the study, how data would be stored and used and highlighted that respondents could stop the interview at any time. We confirmed that the script was read and whether respondents gave consent to continue within ODK on the android phone. We did not ask for written consent as felt that this was not appropriate in the informal setting of the zoo, and would potentially off-put respondents. No names of respondents were collected and all data was saved on a password protected computer. Individual respondents were not informed that the donation at zoo entrance was linked to the choice experiment. Barcode information gave us only the ticket type (e.g. adult, concession) and donation amount, no personal details of the respondents could be obtained (e.g. no bank or card details or personal names).

### Data analysis

Final choice tasks were analysed using R (version 3.2.2) and included in a mixed multinomial logit model (MIXL) in the GMNL package [49]. To allow identifiability, the model was specified so that the probability of selecting a conservation management scenario was a function of attributes of that scenario and of the alternative specified constant (ASC). The ASC captures the effects of utility of attributes not included in the choice specific attributes [41]. In this case the ASC estimates the utility for the baseline project relative to BAU and was coded 0 for BAU and 1 otherwise. When the parameter estimates are obtained by the use of the MIXL model, welfare measures, in the form of willingness to pay, can be determined by estimating the change in the conservation management attribute in question and the utility of income represented by the coefficient of the cost attribute.

While unobserved heterogeneity can be accounted for in the MIXL base model, the model fails to explain the sources of heterogeneity [50]. By including interactions with respondent-specific socio-economic data with choice specific attributes, the model can identify variations in random and conditional heterogeneity in choice preferences. Socio-demographic details of respondents were included as dummy variables into the final model. The income variable was adjusted for co-habiting respondents and was dummy coded for above average household income in the UK at £23,556 per annum [51]. We created a dummy coded variable for high education, where respondents having a degree, or post graduate degree were given a 1, all others a 0. We also created dummy variables as to whether the respondent had previously donated to: any charity, a wildlife charity or a humanitarian charity (coded as a 1 for donate and 0 for not donating)

We then wanted to analyse the interactions between the revealed preference study and the choice experiment. Firstly we used a chi-squared test to test whether the proportions of individuals donating or refusing differed depending on campaign type. We then hypothesised that those exposed to the threatened species management campaign would have higher preference to the threatened species population attribute within the choice experiment. All respondents within the choice experiment survey had been exposed to one of the two marketing campaigns, therefore we created a dummy variable for which marketing campaign the choice experiment was conducted under (related to the date of the survey). For “exposure to species campaign” those respondents that were exposed to the threatened species campaign were given a 1 and those exposed to the community involvement in management campaign a 0. This allowed us to analyse the effects of marketing exposure on preferences within the choice experiment. These exposure variables were only interacted with two of the attributes within the choice experiment: threatened species populations and community involvement in management.

Due to a limited number of respondents that ended up both specifically being asked for a donation, and participating in the choice experiment (due to the random sampling) we ran a donor base MIXL model using only those individuals that gave a real donation during either campaign. Finally we wanted to test two hypotheses on the difference between donors and refusers. The first hypothesis was that those that refused to give a donation, under either campaign would have a lower stated willingness to pay than those that gave a real donation for both the species populations attribute and the community involvement in management attribute. Secondly we tested the hypothesis that those that refused to donate in the real campaigns would have a more negative payment coefficient due to refusing to give a donation in real life.

We used the base MIXL model of all respondents to the choice experiment. We identified the responses of those individuals that had given a donation to either campaign, and those that had refused to give a real donation in either campaign. We extracted the individuals’ conditional mean willingness to pay for the two attributes within the choice experiment and also extracted the parameter coefficient for the payment attribute using the conjoint package in R. We then conducted a series of t-tests to see if those that refused to donate had significant difference in their willingness to pay for either the threatened species populations attribute or community involvement in management attributes.

## Results

### Descriptive statistics

**Fig 2:**
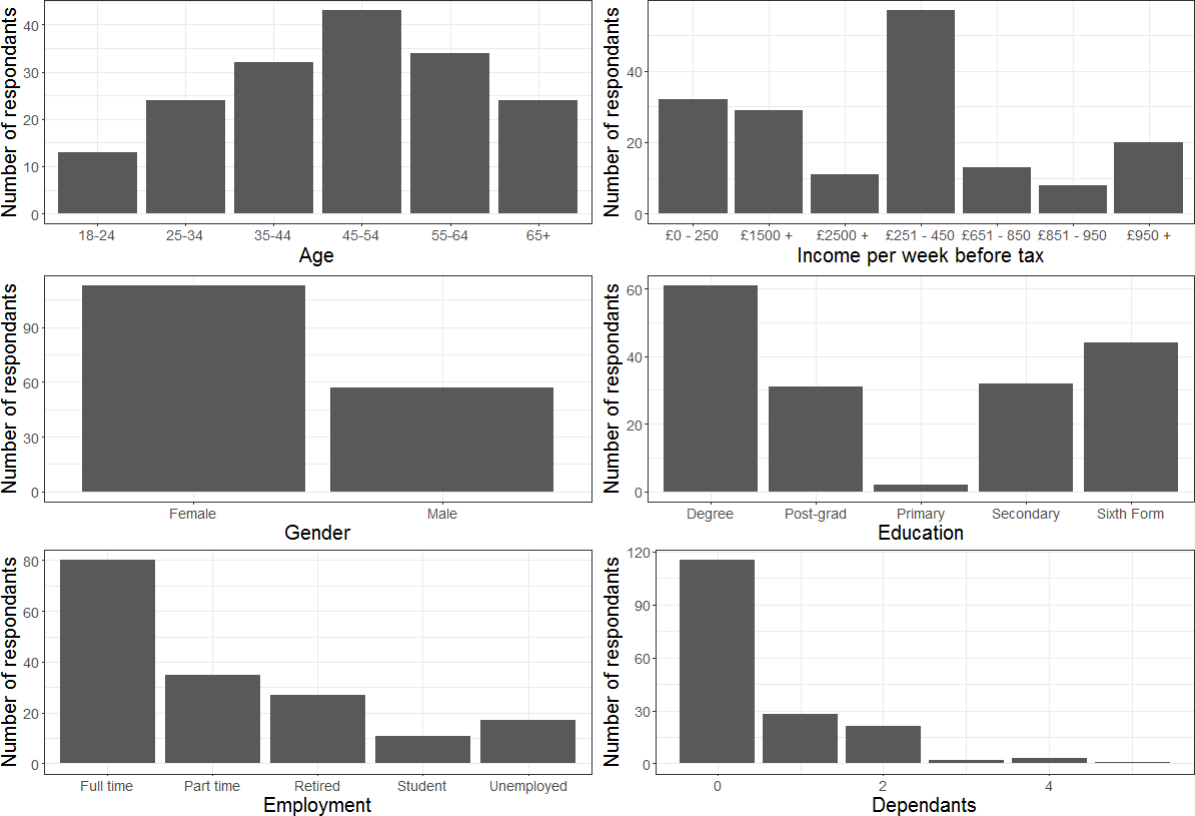
Descriptive statistics of the respondents within the choice experiment survey.

A total of 244 choice experiment interviews were conducted with an additional 31 refusals and 10 individuals that dropped out during the interview. We have limited socio-demographic data of the paying footfall in the zoo but a comparison on the data we have from till sales during our study period suggests that we achieved a relatively similar proportion of student, adult and retired respondents (see Table B in S2 Appendix). The results show a relatively even distribution across age groups, though more females were interviewed than males and results are skewed towards those with less children (Fig 2).

### MIXL model and interactions

From the 244 completed surveys, after non responses were excluded, 1505 choices were included in a MIXL base model (Table A in S3 Appendix). The negative sign on the payment coefficient, shows that respondents prefer options that cost less, which is in line with expectations. The remaining attributes are of the positive and are all highly significant at the 1% level suggesting a focus on threatened species populations, community involvement in management, condition of conservation sites and alternative livelihood investment are all valued as part of a conservation project by donors. The mean coefficients for the attributes threatened species populations and community involvement in management are much higher than the other attributes within the base model. Therefore, visitors to the zoo appear to derive particular utility from (and therefore have a stronger preference for) those conservation projects that improve threatened species populations in Madagascar and incorporate community involvement in management. The positive sign on the alternative specific constant (ASC) coefficient shows that respondents also prefer a project incorporating all the base level attributes compared to BAU.

Gender of respondent (n = 69 males) and having a child under the age of 18 (n = 42) had no significant effect on stated preferences for any of the attributes within the choice experiment. We also found that a respondent’s previous donations to wildlife charities, humanitarian charities or both had no significant effect on which attribute was chosen. This implies previous charitable donation to a humanitarian charity had no impact on respondent stated preference for either providing alternative livelihoods or community involvement in management in Madagascar.

We tested a series of socio-demographic variables within the model, only three variables improved model fit: above average income, degree or graduate level education and exposure to the species campaign (Table B in S3 Appendix, for results of the interacted MIXL model). These were interacted against all of the attributes in a series of models, but the best fit occurred when only interacted with the threatened species populations attribute. This increased the log likelihood from -972 in the base model to -968 and decreased the AIC value from 1967 to 1965. Those respondents with higher education had a significantly higher preference for threatened species populations improving than those with lower education. Those respondents with higher than average income also tended to have positive preferences for this attribute, though this interaction was not significant.

We tested the hypothesis that those exposed to the threatened species management campaign would have higher preference to the threatened species population attribute within the choice experiment. We interacted the dummy coded “exposure to species campaign” with the threatened species populations attribute (Table B in S3 Appendix). The sign for the interacted variable was negative. This implies that some respondents exposed to the threatened species management campaign tended to have lower preference for the threatened species population attribute but this difference was not significant. The marketing exposure apparently had no effect on the preferences of respondents within the choice experiment itself.

### Marginal willingness to pay

**Fig 3:**
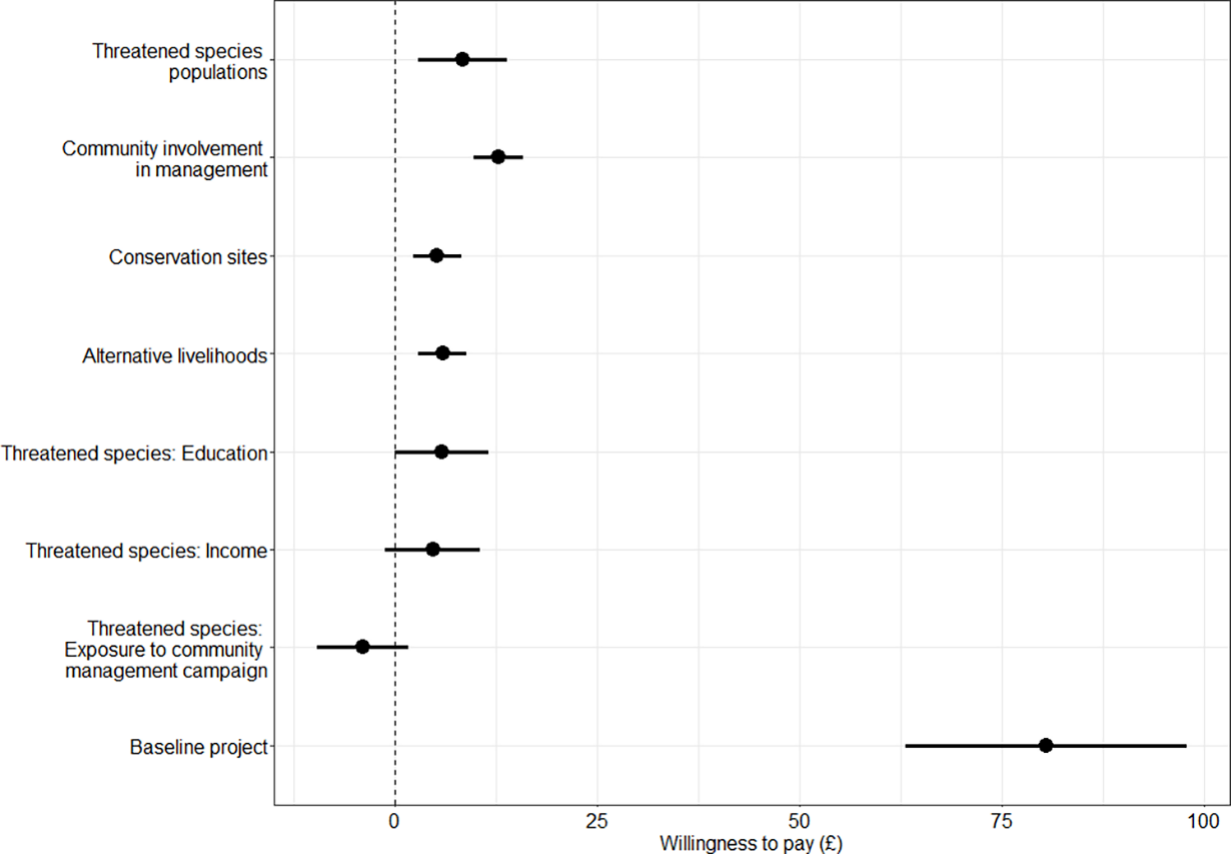
Mean willingness to pay (and 95% confidence intervals) for attributes and interacted socio-economic variables of respondents for conservation management scenarios in Madagascar.

Once the MIXL has been estimated, the parameter estimates can be used to calculate marginal willingness to pay values for each attribute. Fig 3 shows mean willingness to pay (and 95% confidence intervals) for a conservation scenario with high levels of the attributes. The attribute with the highest mean willingness to pay is community involvement in management, followed closely by the threatened species populations attribute. Providing alternative livelihoods and improving conservation sites have similar (lower) support.

Respondents are willing to pay, on average £12.83 (£9.82-£15.84 95% CI) for a community involvement in management programme in Madagascar, compared with the £8.41 (£2.96-£13.87) to improve threatened species populations. There was evidence for positive willingness to pay for both conservation sites (£2.36- £8.21) and providing alternative livelihoods (£3.01–£8.84). The interacted variables with a negative WTP imply that some respondents in that demographic group had a negative preference for those attributes however, this was not significant.

### Revealed preference results

A total of 14,116 paying visitors entered the zoo during the 4 week period. However, due to the volume of visitors entering through the tills, time and personnel constraints, only 13% of these were asked to give a donation (see Table 2 for a summary of the campaigns and the number of donators and refusers). The majority (88%) of those who were asked to donate did make the additional donation (see Table 2). However a chi-squared test showed that there was no significant difference between the proportions that donated or refused under the two marketing campaigns (presented in Table 2).

**Table 2:** Summary results of donators and refusers the two marketing campaigns run at Jersey zoo over a four week period of alternating campaign types during July and August 2016.

*Campaign type*	*Community (%)*	*Threatened species (%)*	*Total (%)*
*Donation*	797 (89)	778 (86)	1575 (88)
*Refusal*	98 (11)	125 (14)	223 (12)
*Total*	895	903	1798

Data Source: Durrell Marketing department July- August 2016. Chi squared = 3.02 (P = 0.074)

Prior to approaching for interviewing we did not know who had been asked to make a donation at the tills, and with the relatively low proportion of visitors who were asked, this resulted in only 15% of the respondents that participated in the choice experiment having also been asked to give a donation. Of these 50 respondents that completed both elements of the experiment, 43 gave a donation and 7 refused. Of these, only 40 were included in the final analysis (36 donators, 4 refusals), due to drop outs and non-responses to the parts of the survey.

**Fig 4:**
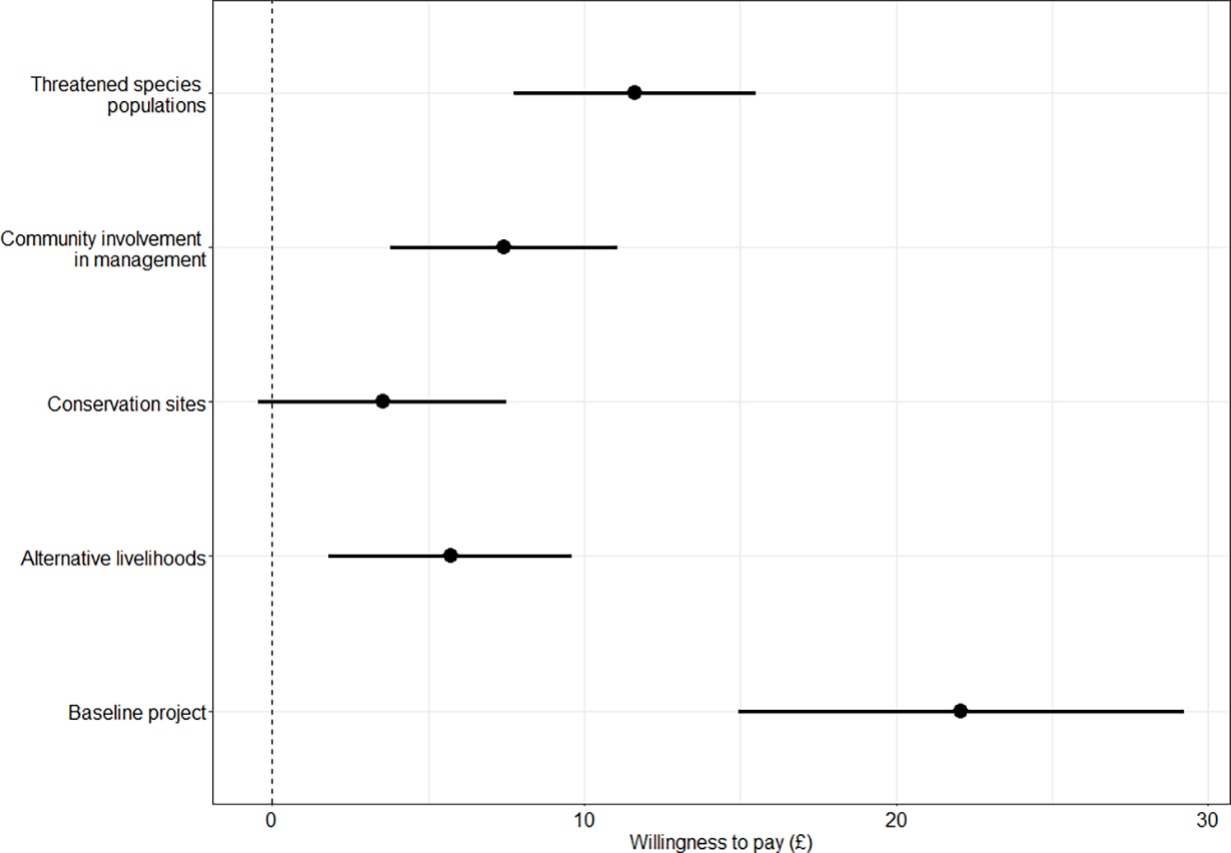
Mean willingness to pay (and 95% confidence intervals) for attributes within the choice experiment for those individuals that gave a real voluntary donation during either marketing campaign prior to participating in the choice experiment.

The MIXL donor base model (with only those individuals that gave a donation during either campaign) showed similar patterns to the model for all respondents but note that the number of observations dropped from 1505 to 318 due to the small sample size. Parameter estimates were used to calculate the marginal willingness to pay for the conservation management attributes. Fig 4 presents the mean willingness to pay and 95% confidence interval for each of the attributes for individuals that gave a real donation during the marketing campaigns. Individuals had a negative preference for an increase in the payment attribute and the two attributes for threatened species populations and for community involvement in management remained significant at the 1% level (Table C in S3 Appendix). The attribute for alternative livelihoods remains significant, but is lower than the community involvement in management attribute or threatened species populations attribute. The attribute for conservation sites is no longer significant. This implies that those that gave a real donation have a stronger stated preference for those conservation projects that improve both threatened species populations and community involvement in management.

**Fig 5:**
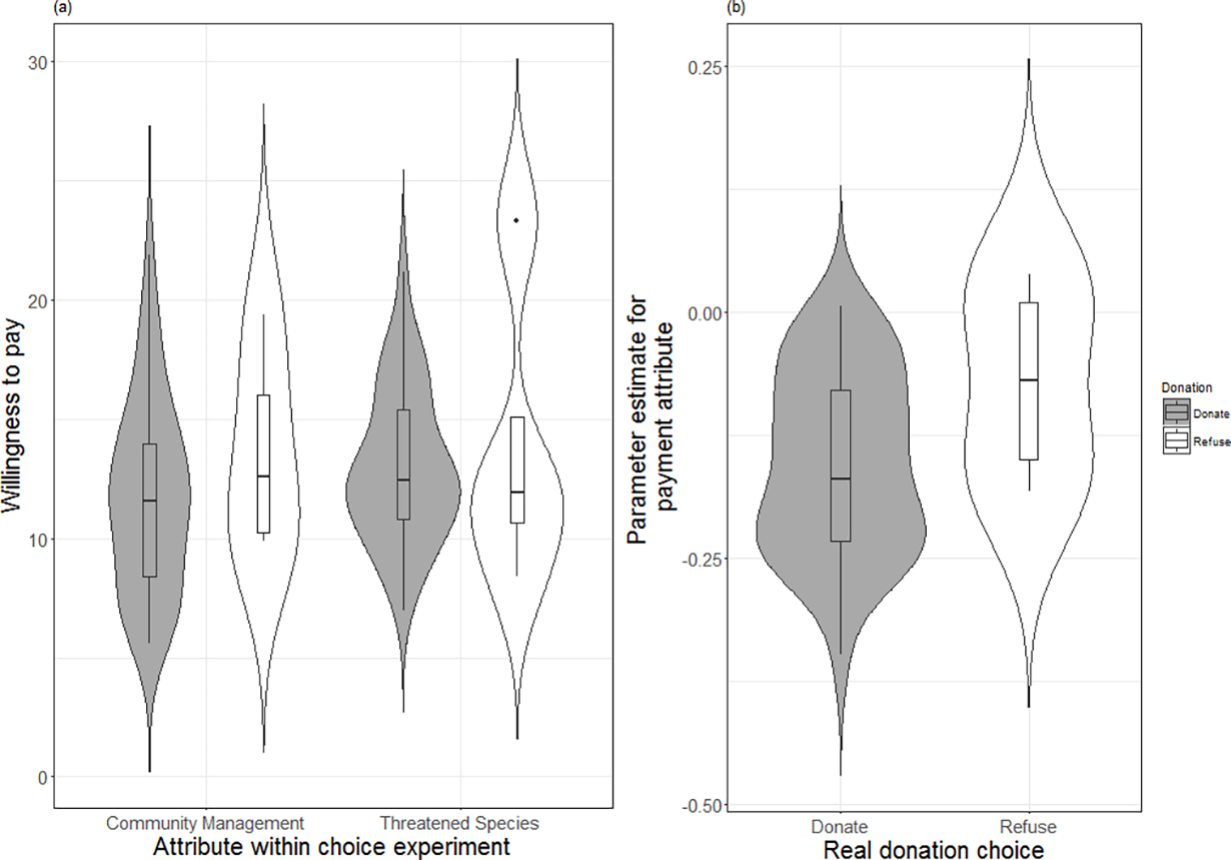
The difference between respondents that donated or refused during the real campaigns for both their willingness to pay and the payment coefficient within the choice experiment. (A) Willingness to pay for respondents that refused or donated in the real campaigns for the corresponding attributes in the choice experiment. Donations and refusals are combined across the marketing campaigns as there was no significant effect of exposure on preference. The violin plots show median, upper and lower quartiles and the centred density. (B) Individual coefficients for the payment attribute within the choice experiment for respondents that either refused or donated within the choice experiment. Donations and refusals are combined across the marketing campaigns as there was no significant effect of exposure.

We tested the hypothesis that those that refused to give a donation, under either campaign would have a lower stated willingness to pay than those that gave a real donation for two of the attributes within the choice experiment; species populations and community involvement in management. The results of the t-tests showed that there was no significant difference between donators and refusers in their stated willingness to pay for either attribute, though the sample size is very small (see Fig 5A). Finally we tested the hypothesis that those that refused to donate in the real campaigns would have a more negative payment coefficient, due to refusing to donate in real life. The sample size was very small for this test and there was no significant difference in the random utilities for the payment attribute within the choice experiment. However, we can see that some individuals refused to give a real donation had positive utilities for the payment attribute (Fig 5B). This implies that some individuals may not have given the payment attribute adequate consideration of their ability to pay and those individuals that gave a real donation tended to have a lower preference for the conservation projects with higher costs.

## Discussion and conclusions

Understanding what potential donors value in a conservation project is necessary to improve the marketing of conservation projects to attract funding, while also revealing insights into how the general public view conservation. We show that visitors to a zoo in Jersey, Channel Islands, have a positive willingness to pay for conservation projects in Madagascar whether focused on delivering improvements in threatened species populations, improving the condition of important sites, involving local communities in management, or providing support for alternative livelihoods. However there was a particularly strong willingness to pay for projects with community involvement in management. This preference was seen even on days when a threatened species-focused marketing campaign was running and when analysing the results of only those that gave a real donation. This implies that emphasising a participatory, community conservation approach to conservation is attractive to potential donors and could increase funding.

Socio demographic characteristics of respondents did not have a significant effect on our results. Our sample did however contain people with less dependants potentially undervaluing the attributes with associated bequest values [52]. If the study had been done after the zoo visit, this may have increased both stated and revealed preference amounts. However the logistics (donations were requested at the cash desk as visitors paid to enter the zoo) meant it wasn’t possible to ask for a donation after the visit and so while it would have been possible to conduct a choice experiment after a respondent’s zoo experience to test for deliberative effects [21], this was not done as we wanted the revealed preference study and the stated preference study to be comparable.

Hypothetical bias is often present in choice experiment studies as respondents do not have to back up their statements with real commitments [53–55]. Many authors have suggested that the reliability and validity of choice experiments should be tested through comparisons with real or simulated markets [22,56]. We attempted to externally validate the findings from a choice experiment using a real marketing campaign on the same sample of respondents. We showed that exposure to the campaign types had no effect on the preference of respondents nor did the real donation to either campaign. We also looked at the effect of campaign type on the willingness to pay of those that donated. The amount of respondents that participated in both elements of the experiment was unfortunately too small to reliably estimate any difference between those that gave real donations compared to those that refused. However, it is interesting that, if anything, those who donated tended to be those with a lower willingness to pay than those who refused. This lends tentative support to those who question the validity of choice experiment due to overstatement of willingness to pay due to the hypothetical nature of stated preference valuation techniques [54,55].

Although we don’t believe that respondents associated the revealed preference donation study (conducted by the zoo at their cash desk), with the choice experiment survey (conducted by researchers), there is of course the potential that those asked to make a donation have considered their willingness to pay more concretely, than those involved in the choice experiment alone without previously being asked for a donation. This could result in differences in hypothetical bias between those asked for a donation and those not asked. Unfortunately, the small number of respondents to the choice experiment who had been asked for a donation meant that we could not explore this effect.

Our sample was of members of the public visiting a particular UK zoo. It would certainly be valuable to carry out further studies exploring preferences for different conservation approaches among the general public both in donor countries, and the countries where such conservation projects are conducted. The insights presented here and the methodology (allowing validation of the choice experiment results with a revealed preference approach) suggest how such research could be carried out.

We collected no qualitative information which might help explain the preferences we observed. However conservation involving community management may be viewed as more legitimate and fair [23]. It may also reflect pragmatic views that conservation which includes local people in management will be more effective, though evidence to support this is mixed [57].

The pilot indicated that respondents considered the attributes as independent of one another and the clear ranking of WTP for the attributes enabled us to treat them as distinct. Conservation projects often are faced with trade-offs and may not able to prioritize all potential approaches at the same time; for example tackling illegal hunting to address reductions in a threatened species may be prioritized over general habitat protection. Choice experiment design requires a trade-off between eliciting the maximum information from respondents, without overburdening them with multiple attributes and choice tasks. Further understanding of what donors prefer in community conservation projects would benefit charitable marketing campaigns.

Areas of high biodiversity often overlap with areas where poverty is widespread [3] and there is also a growing body of research which supports the idea that conservation should be participatory and involve local communities in management [58–60]. Our choice experiment suggested there was overwhelming support for conservation projects in Madagascar incorporating community involvement in management. There is widespread agreement among conservationists working in the country that conservation should include local people as full partners [37,42,61]. It is encouraging that this approach is valued by potential donors.

## Supporting information

S1 Fig Attribute explanation card. (TIF)

S2 Fig Practice choice card. (TIF)

S1 Appendix Oral consent script and attribute explanation. (DOCX)

S2 Appendix Table A. Campaign dates during the choice experiment field experiemnt during August 2016.
Table B. Respondents and total paying visitors to the Zoo based on ticket sales the four week experimental period in July/ August 2016.
(DOCX)

S3 Appendix Table A. results of the MIXL base model with all respondents.
Table B. Results of the MIXL model with socio- economic and marketing exposure interactions (standard error in parenthesis).
Table C. Results of the MIXL base model conducted only with the sample of respondents that gave a real donation during either marketing campaign.
(DOCX)

S4 Appendix Excel file containing metadata, summary, variable names, results and the survey code for android phones. (XLSX)
