# Supplementary figures and images for: To what extent do potential conservation donors value community-aspects of conservation projects in low income countries?
Source: PLoS One. 2018 Feb 16;13(2):e0192935. doi: 10.1371/journal.pone.0192935 (PMC5815612; doi:10.1371/journal.pone.0192935)

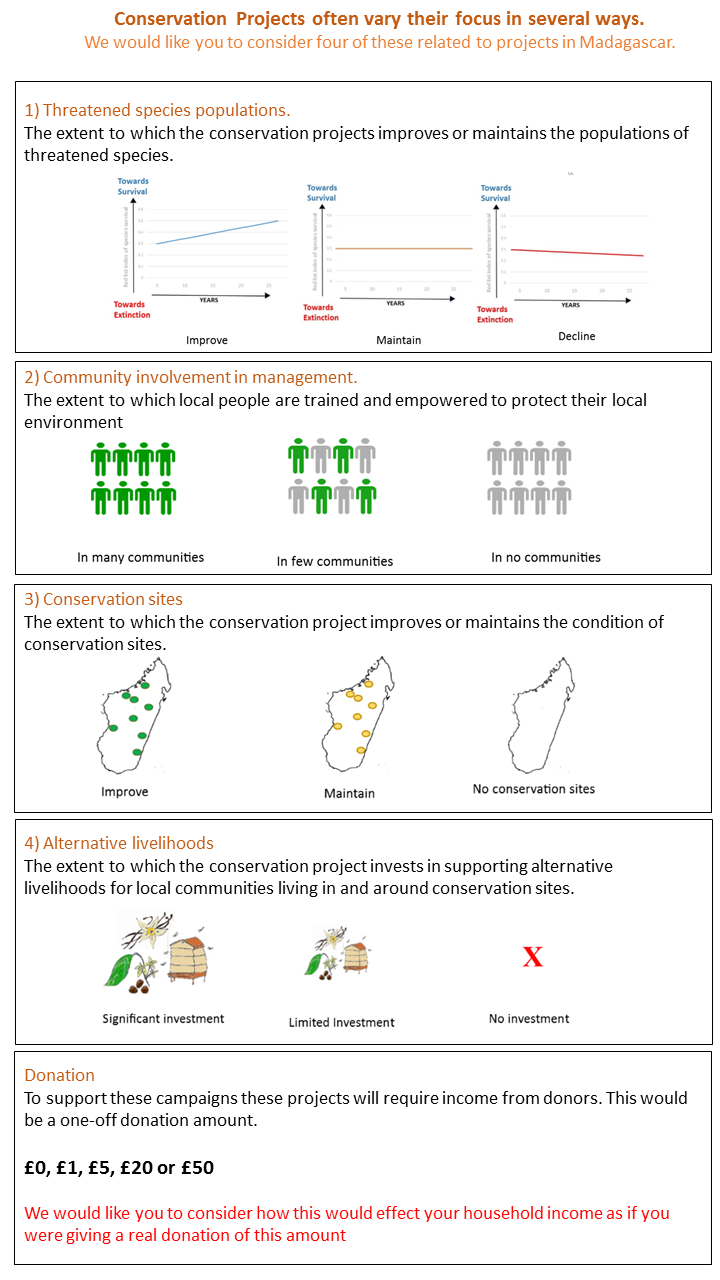

Supplement: S1 Fig — (TIF) [file pone.0192935.s001.tif]

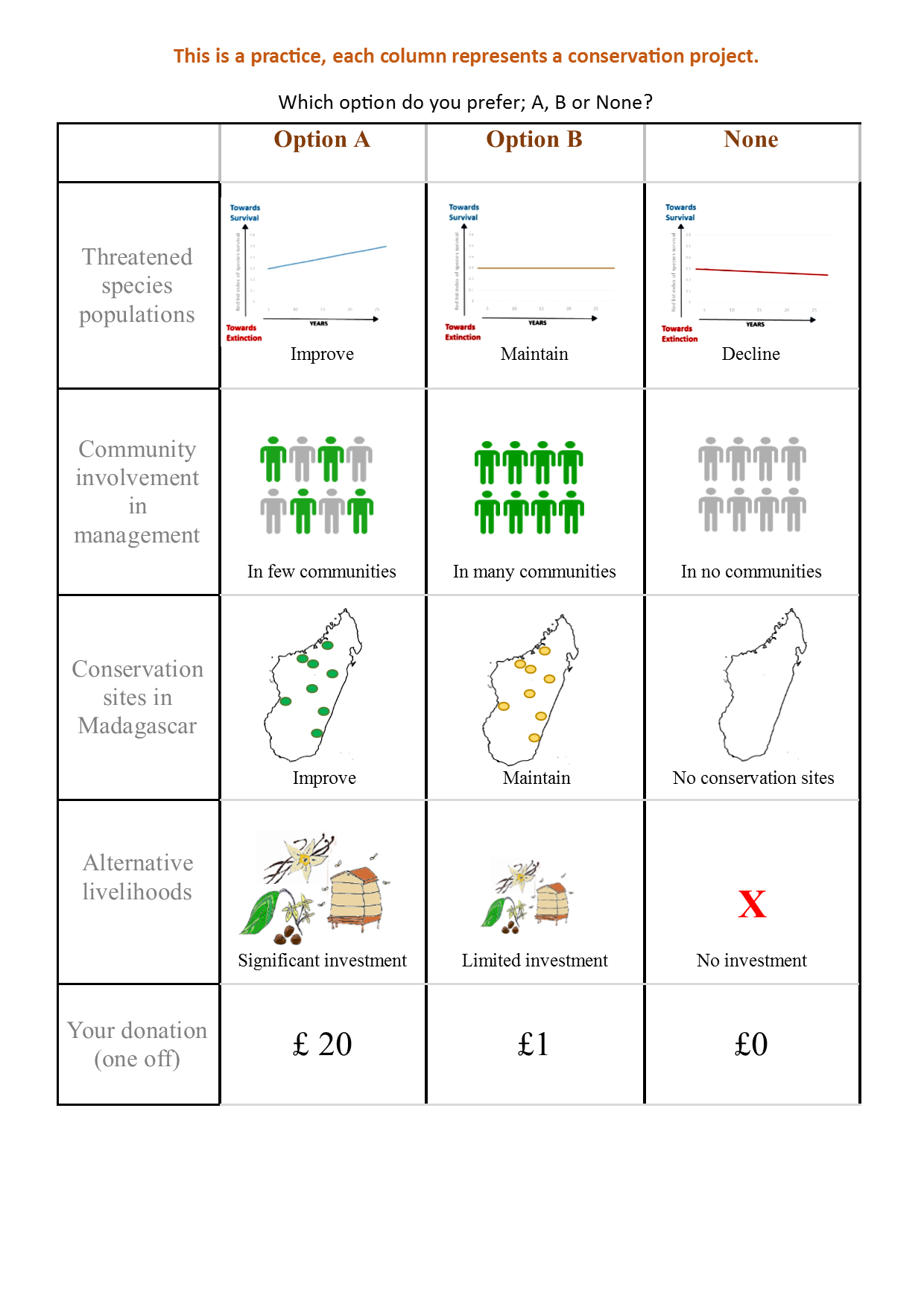

Supplement: S2 Fig — (TIF) [file pone.0192935.s002.tif]
