# Supplementary material for: To what extent do potential conservation donors value community-aspects of conservation projects in low income countries?
Source: PLoS One. 2018 Feb 16;13(2):e0192935. doi: 10.1371/journal.pone.0192935 (PMC5815612; doi:10.1371/journal.pone.0192935)
Supplement: S1 Appendix — (DOCX) [file pone.0192935.s003.docx]

**S1 Appendix. Oral consent script and attribute explanation**

**Oral consent form**

[To be used for Jersey Wildlife Park visitors. Consent form should be read out at the start of the exercise and consent recorded.]

**Introduction to the Research**:

We are researching how conservation projects can best deliver their conservation strategy and what donors perceive as priorities for the areas in which they work. We hope to conduct questionnaires with visitors to the wildlife park to understand if there are any differences based on people’s socio-economic backgrounds. Households are under no obligation to participate in our research. This research is being conducted by an MSc and PhD student at Bangor University, Wales. We are working with several NGOs on this project; Wildfowl and Wetlands Trust (WWT) and Durrell Wildlife Conservation Trust (DWCT).

The interview will take about 30 mins but you are free to stop it at any time. Before we start we want to make sure that you understand the research we are doing and what we will do with the information we collect.

**Oral Consent Script**

1. Did we make things clear? Do you want to ask us any questions about the study?

2. We will keep all the information you give us confidential as far as the law allows. Any notes or recordings we make will be kept on a password-protected computer. We will not share your personal details or personal views with anyone else. Is that okay?

3. Some of the information you give us may be published, but your real name will not be used in relation to any of the information you have provided us, unless you tell us clearly that you want us to use your real name. Is that okay?

4. You should know that even though we will avoid including identifying information in any publication, there is still a possibility that people will recognise you by the things you say. If at any time you feel concerned about what you are saying being disclosed, please feel free to stop and talk to us about it. If you say something that you later think should be deleted from our discussion notes, just let us know. Is that clear?

5. If you mention anything you do not want us to publish, please say so and we will follow your request. Okay?

6. You can stop this interview at any time, without giving us any reason. Okay?

7. We would like to record this interview with a digital audio recorder. That way we can listen to the recording afterwards and catch things you say that we might not fully understand during the interview, or might otherwise forget. Only people in our study team will be able to listen to the recording. Do you give us permission to record?

8. [If appropriate] If you agree, we would like to take some photos. We might use these in presentations or publications about this project. Is this okay?

9. Do you have any further questions? Can we start the interview now?

{Insert Fig S1}

Fig S1. Attribute explanation card.

{Insert Fig S2}

Fig S2. Practice choice card.
