# Supplementary material for: To what extent do potential conservation donors value community-aspects of conservation projects in low income countries?
Source: PLoS One. 2018 Feb 16;13(2):e0192935. doi: 10.1371/journal.pone.0192935 (PMC5815612; doi:10.1371/journal.pone.0192935)
Supplement: S2 Appendix — Table A. Campaign dates during the choice experiment field experiemnt during August 2016. Table B. Respondents and total paying visitors to the Zoo based on ticket sales the four week experimental period in July/ August 2016. (DOCX) [file pone.0192935.s004.docx]

**S2 Appendix**

Table A. Campaign dates during the choice experiment field experiemnt during August 2016.

| Week | Date in August 2016 | Campaign type |
| --- | --- | --- |
| 1 | 01 - 07 | Community involvement in management |
| 2 | 08 - 14 | Threatened species populations |
| 3 | 14 - 21 | Community involvement in management |
| 4 | 22 - 28 | Threatened species populations |

Table B: Respondents and total paying visitors to the Zoo based on ticket sales the four week experimental period in July/ August 2016.

|  | Paying visitors (%) | Respondents (%) |
| --- | --- | --- |
| Student | 533 (4.6%) | 15 (6.2 %) |
| Adult | 8097 (69.4%) | 172 (70.4%) |
| Retired | 3031 (26%) | 57 (23.3%) |
| Total | 11661 | 244 |
| Data source: Durrell Wildlife Conservation Trusts and DCE survey 2016. | | |
