# Supplementary material for: To what extent do potential conservation donors value community-aspects of conservation projects in low income countries?
Source: PLoS One. 2018 Feb 16;13(2):e0192935. doi: 10.1371/journal.pone.0192935 (PMC5815612; doi:10.1371/journal.pone.0192935)
Supplement: S3 Appendix — Table A. results of the MIXL base model with all respondents. Table B. Results of the MIXL model with socio- economic and marketing exposure interactions (standard error in parenthesis). Table C. Results of the MIXL base model conducted only with the sample of respondents that gave a real donation during either marketing campaign. (DOCX) [file pone.0192935.s005.docx]

**S3 Appendix**

Table A. results of the MIXL base model with all respondents

| Attributes and Interactions | MIXL. Base model (S.E) |
| --- | --- |
| ASC | 4.13 (0.444)*** |
| Payment | -0.051 (0.004)*** |
| Threatened species populations | 0.608 (0.080)*** |
| Conservation sites | 0.270 (0.001)*** |
| Alternative livelihoods | 0.269 (0.076)*** |
| Community involvement in management | 0.655 (0.083)*** |
| Log Likelihood | -972.31 |
| AIC | 1966.619 |
| Observations | 1505 |
| Note: *** 1% significance level, ** 5% significance level, *10% significance level with two-tailed tests. The positive sign of the ASC shows that respondents prefer options that move away from the BAU scenario. | |

Table B. Results of the MIXL model with socio- economic and marketing exposure interactions (standard error in parenthesis).

| Attributes and Interactions | MIXL model with interactions (S.E) |
| --- | --- |
| ASC | 4.07 (0.444)*** |
| Payment | -0.05 (0.003)*** |
| Threatened species populations | 0.43 (0.143)** |
| Conservation sites | 0.27 (0.076)*** |
| Alternative livelihoods | 0.30 (0.076)*** |
| Community involvement in management | 0.65 (0.082)*** |
| Threatened species populations: education | 0.30 (0.151)* |
| Threatened species populations: income | 0.24 (0.151) |
| Threatened species populations: exposure to species campaign | -0.20 (0.146) |
| Log Likelihood | -968 |
| AIC | 1965 |
| Observations | 1505 |
| Note: *** 1% significance level, ** 5% significance level, *10% significance level with two-tailed tests. Exposure was calculated based on the date the survey took place and which marketing campaign was running (“community involvement in management” or “threatened species management”). The positive sign of the ASC shows that respondents prefer options that move away from the BAU scenario. | |

Table C. Results of the MIXL base model conducted only with the sample of respondents that gave a real donation during either marketing campaign.

| Attributes and Interactions | MIXL Base model (S.E) with donators to either marketing campaign |
| --- | --- |
| ASC | 2.490 (0.497)*** |
| Payment | -0.113 (0.016)*** |
| Threatened species populations | 1.314 (0.307)*** |
| Conservation sites | 0.400 (0.224) |
| Alternative livelihoods | 0.645 (0.234)** |
| Community involvement in management | 0.839 (0.242)** |
| Log Likelihood | -177.59 |
| AIC | 377.19 |
| Observations | 318 |
| Note: *** 1% significance level, ** 5% significance level, *10% significance level with two-tailed tests. Results from this model represent only those respondents that completed the choice experiment and that either donated or refused in the real campaigns. This is coded as 1 if donated to the community management campaign | |
